# Vocal Fry May Undermine the Success of Young Women in the Labor Market

**DOI:** 10.1371/journal.pone.0097506

**Published:** 2014-05-28

**Authors:** Rindy C. Anderson, Casey A. Klofstad, William J. Mayew, Mohan Venkatachalam

**Affiliations:** 1 Department of Biology, Duke University, Durham, North Carolina, United States of America; 2 Department of Political Science, University of Miami, Coral Gables, Florida, United States of America; 3 Fuqua School of Business, Duke University, Durham, North Carolina, United States of America; UNLV, United States of America

## Abstract

Vocal fry is speech that is low pitched and creaky sounding, and is increasingly common among young American females. Some argue that vocal fry enhances speaker labor market perceptions while others argue that vocal fry is perceived negatively and can damage job prospects. In a large national sample of American adults we find that vocal fry is interpreted negatively. Relative to a normal speaking voice, young adult female voices exhibiting vocal fry are perceived as less competent, less educated, less trustworthy, less attractive, and less hirable. The negative perceptions of vocal fry are stronger for female voices relative to male voices. These results suggest that young American females should avoid using vocal fry speech in order to maximize labor market opportunities.

## Introduction

Vocal fry, also known as glottalization, pulse phonation, or “creaky voice,” refers to a quality of voice characterized by intermittent irregular vibrations of the vocal folds (i.e., vocal cords) in the larynx (i.e., voice box). More specifically, vocal fry is produced through brief glottal pulses followed by vocal fold adduction [1], [2], resulting in a voice quality accompanied by creaking, cracking, and popping noises [3], [4], [5]. This quality of speech occurs typically when speakers lower their vocal pitch to the lowest register they are capable of producing [3], [4]. Vocal fry can be associated with a speech pathology or produced voluntarily as a vocal affectation [3], [4], [5]. Recent research has noted a high proportion of young adult females in the United States using vocal fry, particularly when vocalizing words occurring at the end of utterances [5], [6]. The habitual use of vocal fry speech patterns has sparked debate over whether the affectation enhances or degrades perceptions of this demographic, particularly in the eyes of employers.

Some forward that young American women who speak with vocal fry are perceived as educated and upwardly mobile [6], which would imply favorable outcomes in the job market. Supporters of this view refer to the use of vocal fry among successful females working in traditionally male dominated industries such as finance [7] and print media [8], suggesting vocal fry as a means of communicating seriousness, intelligence, and determination. As vocal fry typically occurs when speakers lower their vocal pitch [3], [4], this interpretation comports with research showing that men and women with lower pitched voices are perceived as stronger and more dominant [9], [10], [11].

Others contend that the use of vocal fry is a vocal “fashion trend” that can be off-putting to older generations, in turn damaging the professional image of young women [12], [13]. Some voice coaches who consult business professionals on appropriate speech habits in the workplace advise against the use of vocal fry, forwarding that it is not perceived as authoritative or serious [14], but rather as immature and uneducated [15]. Given that vocal fry typically occurs when speakers lower their vocal pitch [3], [4], this interpretation aligns with research showing that women with lower pitched (i.e., “masculine”) voices are perceived as less attractive [16], [17].

Which of these competing views better characterize how listeners generally perceive vocal fry is unclear, but is important to understand as young American adult females already face sizable challenges in the labor market. Lack of experience due to age, a poor economic environment, and sex discrimination are all barriers to labor market success for this demographic. Given this context, if vocal fry is perceived negatively, young American females would be best advised to avoid its use.

The only existing research on how speakers of vocal fry are perceived that we are aware of is by Yuasa [6] who examined how 175 American undergraduate college students at two universities perceived vocal fry exhibited by one college-aged American woman. Listeners' perceptions of this voice were generally favorable. However, such perceptions may not reflect those of older individuals, those in the very demographic likely to evaluate and hire young American women in the corporate world. Moreover, the Yuasa [6] study was limited to subjects from two universities suggesting the need for further research to assess the generalizability of the findings to a larger geographic footprint. Additionally, the use of one female voice as the vocal stimulus invites the possibility that the idiosyncratic nature of that voice influenced listeners' perceptions. Here we provide more systematic and generalizable evidence on how vocal fry is perceived.

## Materials and Methods

### Vocal Stimuli

In line with Fraccaro et al. [16] our stimuli were produced by speakers mimicking the vocal fry affectation. More specifically, seven young adult females (average age 24 years, range 19–27 years) and seven young adult males (average age 26 years, range 20–30 years) were recorded speaking the phrase, “Thank you for considering me for this opportunity” in both their normal tone of voice and in vocal fry. We chose this particular phrase as it is one we expect would be spoken by a job candidate when attempting to secure employment.

An alternative research design would have been to use naturally occurring instances of vocal fry and normal voice from different speakers since individuals either do or do not tend to use the affectation. We chose not to use this approach because variation in myriad acoustic features of the different speakers' voices other than vocal fry would be difficult to account for as confounding effects. Another option, in line with many studies on perception of voice pitch [18], would have been to present participants with pairs of voices from the same speakers, one in a normal tone of voice and the other manipulated digitally to generate vocal fry. However, to the best of our knowledge there is no existing method to generate such a manipulation.

Before recording the stimuli the investigators described the concept in detail to the speakers. The investigators also provided the speakers with multiple examples of vocal fry by mimicking the affectation, and in videos posted on the Internet. The speakers practiced speaking in vocal fry to hone their ability to accurately produce the affectation prior to recording. The stimuli were recorded as .wav files using a Shure SM57 microphone and a Marantz PMD660 solid state recorder (files available in Supporting Information). Each audio file was inspected aurally and visually in Audacity (version 2.0.1) to ensure that they were free from speech errors and non-speech noise. Amplify in Audacity (version 2.0.1) was used to normalize the peak amplitude (i.e. “loudness”) of the recordings to 3 dB SPL.

### Participants and Procedures

The experiment was administered online to 800 listeners (400 men and 400 women) by Qualtrics, an online portal for researchers to develop and administer survey questionnaires through the Internet. Online voice perception experiments have been shown to produce results that are comparable to laboratory experiments [19]. Furthermore, Qualtrics can reach a larger and more nationally representative sample of participants than a sample of college students. For this experiment Qualtrics partnered with Survey Sampling International (SSI) to recruit the participants. SSI maintains panels of subjects that are only used for research. Individuals voluntarily join a SSI panel by responding to an online SSI advertisement (e.g., a banner advertisement on a website). Participants received between $.50–.$75 USD in exchange for voluntary participation in this study. They were invited to participate in the study by email. To maximize external validity, Qualtrics drew sample listeners evenly from three age groups: 18–33 (N = 265, 133 women and 132 men), 34–50: (N = 268, 134 women and 134 men), and 51–65 (N = 267 133 women and 134 men).

The experiment was a between-subjects design. Listeners were assigned randomly to listen and judge either the seven male voice pairs (N = 200 male and 200 female listeners) or the seven female voice pairs (N = 200 male and 200 female listeners). We chose not to have listeners respond to both the male and female voices for two interrelated reasons. First, pre-testing of the experiment showed that the length of the task was perceived as too burdensome by the listeners if they were asked to rate vocal stimuli from both sexes. More specifically, listeners became disengaged from the task. Second, easing subject burden was particularly important in this study because, to our knowledge, there is no precedent in the vocal fry perception literature on how easily listeners ascertain the presence of vocal fry in a voice. Without precise priors on effect sizes given the sparseness of the vocal fry perception literature, we chose a research design that would make the manipulation as salient as possible while at the same time easing participant burden.

Before participating, listeners completed a sound check task to ensure that they could hear audio played by the online survey instrument, and indicated whether they listened through computer speakers (N = 706) or headphones (N = 94). Listeners also reported whether they were currently employed, whether they had ever been involved in the hiring and firing of employees, and their current zip code.

**Figure 1:**
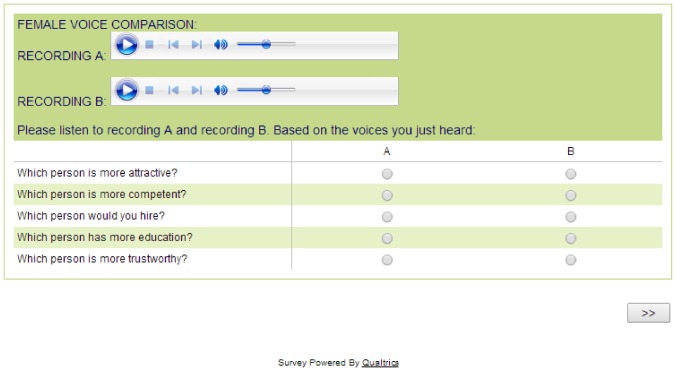
Screenshot of vocal fry perception online assay.

Each pair of audio files was presented on a single web page, and each audio file of a pair was presented separately on the page in its own QuickTime audio player, one labeled “Recording A” and the other labeled “Recording B” (Fig. 1). The audio recordings could be played multiple times. Study participants were instructed to “listen to recording A and recording B.” The order of the seven voice pairs presented to each listener was randomized, as was whether the normal or vocal fry version of each voice was presented first within a pair. This randomization procedure resulted in a counterbalanced design, whereby the vocal fry version of each pair of voices was presented first 49.4% of the time.

After listening to each pair of voices, listeners were asked which speaker was perceived to be more educated, competent, trustworthy, attractive, and which speaker they would hire. The order in which these questions were presented was randomized. We consider education, competence, and trustworthiness as attributes an employer would likely consider when making a hiring decision. We also consider attractiveness because the use of vocal fry has been forwarded as a mechanism to obtain the benefits that accrue to low pitched speech, such as perceptions of social dominance [20] and leadership capacity [18], [21], but also retain femininity [6], [7], [22]. After rating a pair of voices listeners clicked a button with an arrow on it to proceed to a new page with the next pair of voices. Subjects could not proceed to the next page until they completed the five judgments pertaining to each pair of audio files. Listeners' choices were coded as 1 if they selected the fry voice from the pair and 0 if the normal voice was selected. For each listener, the average of choices across the seven voices yields a summary preference ratio ranging from 0 to 1 with higher values indicating a stronger preference for vocal fry.

### Ethics Statement

Prior approval to conduct all elements of the experiment documented in this paper was granted by the Duke University (Durham, North Carolina, USA) Institutional Review Board on August 30, 2013 (Protocol #B0901). Survey Sampling International (SSI), the provider of the research subjects, complies fully with European Society for Opinion and Marketing Research (ESOMAR) standards for protecting individuals' privacy and information. Individuals voluntarily join a SSI panel. All communications between SSI and panel members explain why the member has been selected to participate in a study, what he or she should expect from membership in the panel, and offer multiple methods to opt-out of participating. Study participants provided written consent to participate by voluntarily clicking a link to the survey in the email invitation. Participants were free to stop participating at any time by closing their web browser program. Participation in the study was confidential. Identifying information, such as names or addresses, was not collected during the experiment.

## Results

### Validation of Vocal Stimuli as Vocal Fry Using Acoustic Analysis

**Figure 2:**
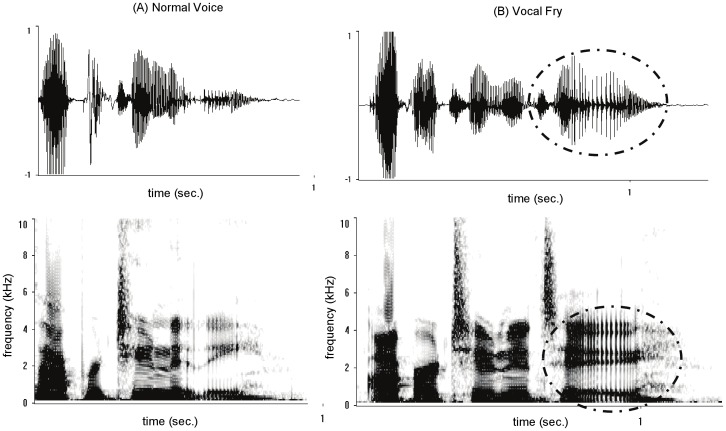
Waveform and spectrogram of the same woman saying the word “opportunity” in normal voice (Panel A) and vocal fry (Panel B). The dashed circles indicate the vibrations in the vocal fry voice at the end of the utterance.

Descriptive statistics for the vocal stimuli, calculated in Praat [23] using the “Get Pitch” command, are presented in Table 1. In line with recommended settings for acoustic analysis in Praat [23], the pitch of the female voices was measured within a range of 100–600 Hz, and for male voices within a range of 75–500 Hz. All other system settings were set to their defaults. Consistent with vocal fry correlating with speakers lowering their vocal pitch to the lowest register they are capable of producing [3], [4], the vocal fry version of each voice is significantly lower in mean F_0_ than the normal voice. We also used Syrinx (version 2.64) to examine the vocal stimuli visually to confirm the presence of vocal fry. Figure 2 shows an example of the spectrogram and waveform from the same woman speaking the word “opportunity” in normal voice (Panel A) and in vocal fry (Panel B). As in Wolk et al. [5] and Yuasa [6], we find that the vocal fry version of each pair of voices is characterized by pulsed amplitude modulations, or “creakiness,” particularly at the end of the utterance as indicated by the dashed circles on the figure.

**Table 1:** Descriptive statistics of acoustic features of vocal stimuli.

	Male Voices (N = 7 pairs)			Female Voices (N = 7 pairs)		
	Normal	Fry	Difference	Normal	Fry	Difference
Mean F_0_ (Hz)	123.86	108.29	−15.57*	204.29	187.57	−16.72*
Min F_0_ (Hz)	81.14	77.29	−3.85∧	105.71	103.14	−2.57*
Max F_0_ (Hz)	224.71	191.14	−33.57	323.57	369.29	45.72
F_0_ Range (Hz)	143.71	113.71	−30.00	218.00	266.00	48.00

Note: The acoustic analysis was conducted in Praat (Boersma & Weenink 2013). Cell entries are averages across the voices.

∧ p≤.06.

*p≤.05,

**p≤.01,

***p≤.001 (Wilcoxon Matched Pairs Test).

### Perceptions of Speech Exhibiting Vocal Fry

**Figure 3:**
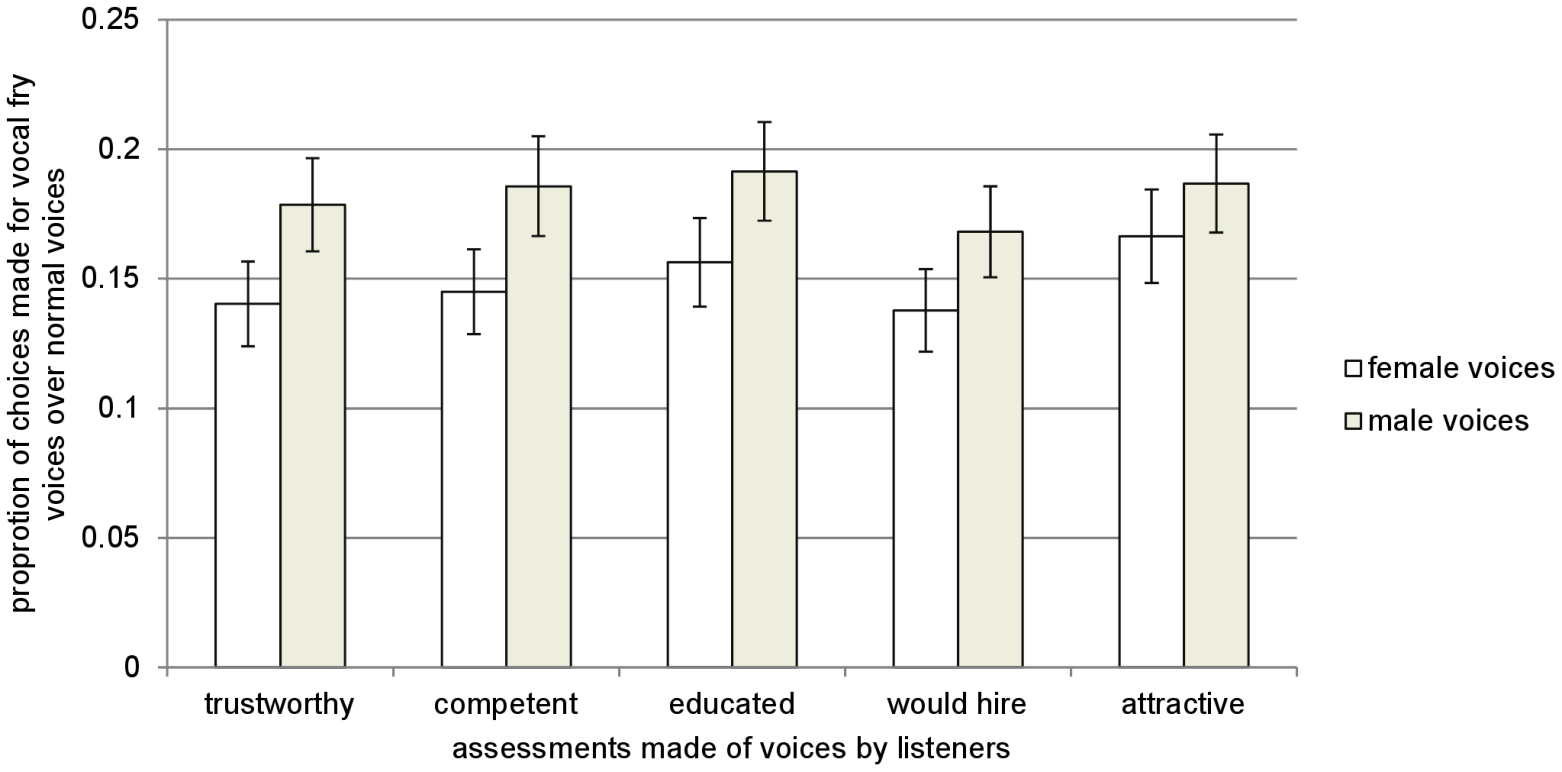
Average preference ratios for vocal fry over normal voice (+/− 95% confidence interval) by sex of speaker. Preference ratios reflect the proportion choices made in favor of vocal fry relative to a normal speaking voice. A preference ratio of 1 (0) reflects a strict preference for vocal fry (normal voice), while 0.50 indicates no discernible preference for either vocal fry or normal voice voices.

The listener is the unit of analysis. Analyses were conducted in SPSS (version 19) and Stata/SE (version 12.1). Two-tailed one-sample t-tests were used to compare listeners' average preference ratios to .50, which represents indifference between a normal voice and vocal fry. As shown in Figure 3, preference ratios for all five assessments by speaker gender are substantially below .50, indicating that a normal voice was preferred by listeners over vocal fry at a rate greater than chance (p<.001).

To test for effects of both sex of speaker and sex of listener, a full-factorial between-subjects analysis of variance (ANOVA) of listeners' preferences was conducted with speaker sex, listener sex, and the interaction of listener and speaker sex. Type of playback device (headphones or computer speakers) was included as a covariate. The results of this analysis show that the negative perception of vocal fry was significantly stronger when listeners judged female voices on trustworthiness (F_1,795_ = 9.54, p<.01, partial η^2^ = 1.19%), competence (F_1,795_ = 10.11, p<.01, partial η^2^ = 1.26%), education (F_1,795_ = 7.24, p = .01, partial η^2^ = 0.90%), and willingness to hire (F_1,795_ = 6.33, p = .01, partial η^2^ = 0.79%), but not in the case of attractiveness (F_1,795_ = 2.33, p = .13). Additionally, while a normal voice is always strongly preferred over vocal fry, female listeners have a more negative perception of vocal fry than male listeners for all five judgments made of the voices (trustworthiness: F_1,795_ = 4.97, p = .03, partial η^2^ = 0.62%; competence: F_1,795_ = 7.50, p = .01, partial η^2^ = 0.93%; education: F_1,795_ = 7.02, p = .01, partial η^2^ = 0.87%; attractiveness: F_1,795_ = 12.57, p<.01, partial η^2^ = 1.16%; willingness to hire: F_1,795_ = 4.97, p = .03, partial η^2^ = 0.62%). While there is statistically significant variation in judgment of vocal fry by sex of the listener and sex of the speaker, the effect sizes (partial η^2^) are small because vocal fry is universally derided regardless of the sex of the speaker or listener. There were no statistically significant interactions between speaker sex and listener sex.

### Heterogeneity in Perceptions of Vocal Fry

Yuasa [6] documents that young adults perceive vocal fry favorably. To assess whether perceptions of vocal fry were more favorable among young adults relative to older listeners, an indicator of whether the listener was between the ages of 18 and 33 (N = 265) was added to the ANOVA analysis described above. The results reveal that while vocal fry is perceived negatively regardless of the age of the listener, older listeners perceived vocal fry more negatively when asked to judge competence (F_1,791_ = 4.03, p = .045, partial η^2^ = 0.52%). We obtained a similar finding for judgments of attractiveness, though this result does not meet the traditional threshold for statistical significance (F_1,791_ = 3.74, p = .053).

To investigate whether the overall negative perceptions of vocal fry are particularly reflective of business leader perceptions, an indicator for whether the listener ever held an employment position in which they hired or fired workplace colleagues (N = 261) was added to the ANOVA analysis. The results show no main effect for being a business leader (trustworthiness: F_1,791_ = .04, p = .84; competence: F_1,791_ = .06, p = .81; education: F_1,791_ = .10, p = .75; attractiveness: F_1,791_ = 2.56, p = .11; willingness to hire: F_1,791_ = .18, p = .68). The interaction of whether the listener was a business leader and listener sex also shows no statistically significant difference between how critical male business leaders are of vocal fry compared to female business leaders (trustworthiness: F_1,791_ = 2.95, p = .086; competence: F_1,791_ = 2.77, p = .096; education: F_1,791_ = .74 p = .39; attractiveness: F_1,791_ = 3.12, p = .078; willingness to hire: F_1,791_ = 2.52, p = .113).

Yuasa [6] documents favorable vocal fry perceptions based on listeners at two universities, one in Iowa and one in California. To assess whether perceptions of vocal fry are positive in these particular geographic regions an indicator of whether the listener's zip code was in Iowa or California (N = 84) was added to the ANOVA analysis. We observe no differences in perceptions of vocal fry between listeners in these states relative to the rest of the United States (trustworthiness: F_1,791_ = .36, p = .55; competence: F_1,791_ = .09, p = .76; education: F_1,791_ = .03, p = .86; attractiveness: F_1,791_ = .12, p = .74; willingness to hire: F_1,791_ = .03, p = .88).

### Perceptions of Vocal Fry and Employability

In a standard interview setting, an employer will listen to potential candidates, form judgments about the candidate on various dimensions, and ultimately decide whether the candidate should be hired as an employee. To assess which judgments are most important for hiring outcomes, we estimated a linear regression model where listeners' hiring preference is the outcome variable, and perceptions of trustworthiness, competence, education, and attractiveness in response to vocal fry are the independent variables. More specifically, the regression model takes the following form with the listener as the unit of analysis:
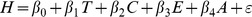
(1)Here each variable represents the proportion of the time participants selected the speaker with vocal fry out of the seven pairs of voices, where *H* is willingness to hire, *T* is trustworthiness, *C* is competence, *E* is education, and *A* is attractiveness. The coefficients in equation (1) capture the implicit weights that the participant placed on each of the speaker attributes when making a hiring decision.

The results of estimating equation (1) for all participants presented in Table 2, Column 1, indicate that while all four vocal perceptions exhibit statistically significant associations with willingness to hire, the influence of perceptions of trust is stronger than that of perceptions of competence (*β_1_*>*β_2_*, F_1,795_ = 21.71, p<.001), education (*β_1_*>*β_3_*, F_1,795_ = 29.43, p<.001), and attractiveness (*β_1_*>*β_4_*, F_1,795_ = 114.24, p<.001). The influence of perceptions of competence and education are not significantly different (*β_2_* = *β_3_*, F_1,795_ = .72, p = .40), but both have greater influence relative to perceptions of attractiveness (*β_2_*>*β_4_*, F_1,795_ = 38.04, p<.001; *β_3_*>*β_4_*, F_1,795_ = 24.39, p<.001). Taken together, these results suggest that job candidates who use vocal fry are not preferred particularly because they are perceived as untrustworthy.

**Table 2:** Relationship between listeners' perceptions of vocal fry and willingness to hire.

	(1) All Observations	(2) Male Listeners/Male Speakers	(3) Female Listeners/Male Speakers	(4) Male Listeners/Female Speakers	(5) Female Listeners/Female Speakers
Trustworthy	.44*** (.03)	.41*** (.06)	.43*** (.06)	.41*** (.04)	.58*** (.05)
Competent	.24*** (.02)	.21*** (.04)	.23*** (.05)	.39*** (.05)	.22*** (.05)
Educated	.21*** (.02)	.28*** (.05)	.13** (.05)	.12** (.05)	.18*** (.04)
Attractive	.06*** (.02)	.05 (.05)	.15*** (.04)	.04∧ (.02)	.002 (.02)
Constant	−.01 (.003)	−.01 (.01)	−.002 (.01)	−.003 (.01)	−.003 (.004)
F	1293.33***	227.46***	240.23***	452.21***	771.35***
Adjusted R^2^	.87	.82	.83	.90	.94
N	800	200	200	200	200

Note: The dependent variable is the preference ratio for willingess to hire. The independent variables represent the preference ratios for perceptions of trustworthiness, competence, education, and attractiveness. Cells contain unstandardized linear regression coefficients, standard errors in parentheses.

∧ p≤.07,

*p≤.05,

**p≤.01,

***p≤.001.

In columns 2–5 of Table 2 we re-estimate equation (1) separately by sex of speaker and sex of listener to test whether the overall effects documented in Column 1 vary based on the sex of the speaker and the listener. While the relationship between perceptions of trustworthiness and willingness to hire appears to be larger in magnitude when women judge women (Table 2, Column 5), it is not statistically different when compared to the magnitudes in the other regression models (Table 2, Columns 2–4). The association between perceived attractiveness and willingness to hire in the overall sample (Table 2, Column 1) is solely due to the subsamples where the speaker and the listener are of the opposite sex (Table 2, Columns 3–4). Also, the model fit and explained variance are superior in the female speakers models (Table 2, Columns 4–5) compared to the male speakers models (Table 2, Columns 2–3), suggesting that vocal perceptions are a better predictor of hiring preferences for female job candidates.

## Discussion

This paper investigates how vocal fry is perceived when used by young American females in a labor market context. A between-subjects experiment conducted on a national sample of American adults reveals that vocal fry is interpreted negatively relative to a normal speaking voice. Young adult female voices exhibiting vocal fry are perceived as less competent, less educated, less trustworthy, less attractive, and less hirable. The negative perceptions of women who use vocal fry are stronger when the listener is also a woman. Collectively, these results suggest young American women should avoid vocal fry in order to maximize labor market perceptions, particularly when being interviewed by another woman.

That vocal fry is interpreted negatively contrasts with the findings of Yuasa [6], which show a general positive perception of vocal fry use by a peer group of young American judges in California and Iowa. While we find some evidence that the negative perception of vocal fry is somewhat muted among younger listeners, the overall perception remains negative regardless of the listener's age. We also observe no differences in the perceptions of listeners residing in Iowa and California relative to those in the rest of the United States, confirming that the generally negative perceptions of vocal fry do not differ by region. The difference in the results of this study and Yuasa [6] are likely related to differences in research design. A distinct advantage of ours is that we use multiple voice exemplars, thereby avoiding idiosyncratic confounds that may arise when using only one speaker as a stimulus [6]. Our study also represents an improvement over Yuasa [6] because our findings are from a national sample of American adults. As such, our results may differ from Yuasa [6] due to the age difference between the listeners included in the study. Understanding precisely why older listeners are more critical of vocal fry is an important aim for future research, and may further illuminate how personal characteristics such as the acoustic qualities of voice influence the perceptions of young women in the labor market.

Our study finds that vocal fry is perceived negatively in both sexes, by both sexes, regardless of the age of the listener. One explanation for this finding is that humans prefer vocal characteristics that are typical of population norms (i.e., “average”). For example, Bruckert et al. [24] show that male and female voices are judged by men and women as more attractive the closer they are in pitch and timbre to the mean of a sample of voices. That is, average voice qualities are preferred. Thus, it is possible that because vocal fry is accompanied by a dramatic reduction in voice pitch relative to normal speech, fry voices are perceived as less attractive.

Why is vocal fry in women perceived more negatively than in men? One explanation is that the lowering of voice pitch via vocal fry results in a sex-atypical voice pitch modulation for females but sex-typical for males. Fraccaro et al. [16] suggest that deliberate sex-atypical voice pitch modulation (i.e. raising pitch in men and lowering pitch in women) is interpreted negatively in the context of attractiveness. One could therefore view the results here as an extension of this finding to an economic context, whereby deliberate lowering of voice pitch in a sex-atypical manner by females through vocal fry results in negative labor market perceptions.

Although vocal fry is perceived negatively, it appears nonetheless to be increasing in prevalence. One explanation for this pattern is that vocal fry incurs social benefits that do not transfer to the labor market. For example, there may be social acceptance benefits to females to conforming to an increasingly common peer group affectation. Nonetheless, whether such behavior is conscious or unconscious [6], the results here suggest speakers should undertake conscious effort to avoid vocal fry in labor market settings.

A second conjecture is that young American females believe that vocal fry can be used to reap the benefits that accrue to a deep voice. For example, men and women with lower pitched voices have been shown to be more successful at obtaining positions of leadership [18], [21], [25], and are perceived as more dominant [9], [10], [11], [26]. Yuasa [6] states this conjecture explicitly by noting that the lowering of voice pitch may help females “compete with men by taking advantage of the positive attributes associated with low-pitch male voices” (p. 331). Such a conjecture rests on the assumption that speaking at a lower voice pitch via vocal fry yields the same benefits that accrue to an individual with a naturally lower-pitched voice. This assumption needs to be tested explicitly, however, because vocal fry differs from normal speech in ways other than pitch.

While this study is the first to assess how vocal fry is perceived by a national sample of American adults, a potential limitation of our approach is the use of vocal stimuli from speakers who were coached to speak in vocal fry. As we did not have full experimental control over the stimuli, the vocal fry stimuli may have sounded somewhat unnatural because the speakers adopted a vocal affectation that they may not use normally. Consequently, the listeners could have been responding to both vocal fry and to differences between the pairs of voices that are not associated with vocal fry. As an alternative approach, in line with studies on voice pitch perception [18], [21] and the perception of age in speakers' voices [27], future studies might find it possible to manipulate recorded voices digitally to synthesize the sound of vocal fry while not affecting other acoustic qualities of the recorded voices.

In sum, women continue to face challenges in the labor market, and are still underrepresented in the upper echelons of business leadership [28]. It is known that physical characteristics such as height, weight, and age affect how humans are perceived in terms of sexual attractiveness [29], [30] and leadership ability [31], [32]. This study contributes to this growing body of research by showing how vocal fry impacts perceptions of young women in the labor market, and highlights the potential importance of subtle cues embedded in the human voice.

## Supporting Information

Audio S1 **Female vocal fry stimulus, “Speaker 2.”**
(WAV)

Audio S2 **Female normal voice stimulus, “Speaker 2.”**
(WAV)

Audio S3 **Female vocal fry stimulus, “Speaker 3.”**
(WAV)

Audio S4 **Female normal voice stimulus, “Speaker 3.”**
(WAV)

Audio S5 **Female vocal fry stimulus, “Speaker 5.”**
(WAV)

Audio S6 **Female normal voice stimulus, “Speaker 5.”**
(WAV)

Audio S7 **Female vocal fry stimulus, “Speaker 6.”**
(WAV)

Audio S8 **Female normal voice stimulus, “Speaker 6.”**
(WAV)

Audio S9 **Female vocal fry stimulus, “Speaker 7.”**
(WAV)

Audio S10 **Female normal voice stimulus, “Speaker 7.”**
(WAV)

Audio S11 **Female vocal fry stimulus, “Speaker 8.”**
(WAV)

Audio S12 **Female normal voice stimulus, “Speaker 8.”**
(WAV)

Audio S13 **Female vocal fry stimulus, “Speaker 9.”**
(WAV)

Audio S14 **Female normal voice stimulus, “Speaker 9.”**
(WAV)

Audio S15 **Male vocal fry stimulus, “Speaker 10.”**
(WAV)

Audio S16 **Male normal voice stimulus, “Speaker 10.”**
(WAV)

Audio S17 **Male vocal fry stimulus, “Speaker 11.”**
(WAV)

Audio S18 **Male normal voice stimulus, “Speaker 11.”**
(WAV)

Audio S19 **Male vocal fry stimulus, “Speaker 12.”**
(WAV)

Audio S20 **Male normal voice stimulus, “Speaker 12.”**
(WAV)

Audio S21 **Male vocal fry stimulus, “Speaker 13.”**
(WAV)

Audio S22 **Male normal voice stimulus, “Speaker 13.”**
(WAV)

Audio S23 **Male vocal fry stimulus, “Speaker 14.”**
(WAV)

Audio S24 **Male normal voice stimulus, “Speaker 14.”**
(WAV)

Audio S25 **Male vocal fry stimulus, “Speaker 15.”**
(WAV)

Audio S26 **Male normal voice stimulus, “Speaker 15.”**
(WAV)

Audio S27 **Male vocal fry stimulus, “Speaker 16.”**
(WAV)

Audio S28 **Male normal voice stimulus, “Speaker 16.”**
(WAV)
